# Short-Term Effects of Mitigation Measures for the Containment of the COVID-19 Outbreak: An Experience From Northern Italy

**DOI:** 10.1017/dmp.2020.119

**Published:** 2020-04-24

**Authors:** Giovenale Moirano, Maurizio Schmid, Francesco Barone-Adesi

**Affiliations:** Cancer Epidemiology Unit, Department of Medical Sciences, University of Turin, Turin, Italy; BioLab - Engineering Department - Roma Tre University, Rome, Italy; Department of Translational Medicine, University of Eastern Piedmont, Novara, Italy; CRIMEDIM – Research Center in Emergency and Disaster Medicine, University of Eastern Piedmont, Novara, Italy

**Keywords:** epidemics, epidemiologic methods, population surveillance

## Abstract

We evaluated the short-term effects of mitigation measures imposed by the Italian government on the first 10 municipalities affected by Sars-Cov-2 spread. Our results suggest that the effects of containment measures can be appreciated in about approximately 2 wk.

In the past weeks, several countries implemented mitigation measures in the attempt to curb the coronavirus disease 2019 (COVID-19) epidemic. While these interventions proved to be effective in Wuhan (China), it is not clear whether these results are directly applicable to other countries.^1^ We conducted a first evaluation of the effectiveness of such measures in a small Italian area, taking advantage of the fact these mitigation measures were introduced 2 wk ahead the rest of the country. Following the detection of the first cases of COVID-19 in Lombardy, the Italian Government enforced different policies to contain the local outbreaks. On February 23rd, a total lockdown was issued for 10 municipalities in the Lodi Province (Lombardy Region), and the measures included: (a) strict home confinement to the entire population; (b) closure of all the nonessential commercial activities; and (c) mobility restrictions related to the involved municipalities.^2^ These policies were later extended to the whole of Lombardy (March 8th) and to the entire Country (March 9th).

The early implementation of such regulations in these municipalities (hereafter defined as the “red zone”) allows a sufficient observation time to conduct a thorough evaluation of their effect. Specifically, we investigated changes in the time-varying reproductive number, *R_t_*, namely the estimate of the average number of secondary cases that each infected individual would infect if the conditions remained as they were at time, t.^3^ A decrease of *R_t_* over time provides insights to the effectiveness of the interventions, given that the goal of control efforts is to reduce it below the threshold value of 1. Data on incident cases of COVID-19 recorded between February 28th and March 27th were obtained from the website of the Italian Civil Protection.^4^ As data at the municipality level are not available, we used data from the whole Lodi province and 2 neighboring provinces to indirectly estimate the daily number of cases in the red zone. Observed rates in the whole Lodi province are, indeed, a weighted mean between the rates in the red zone (accounting for 22% of the population of the Province) and the rates in the other municipalities (the remaining 78% of the provincial population).

We assumed that the municipalities of the Lodi Province out of the red zone had incidence rates similar to the neighboring provinces (Milan and Pavia). Applying incidence rates of Milan and Pavia provinces to the 78% of Lodi Province population, we could calculate the expected number of new cases occurring in the red zone. We then estimated the time-varying reproductive number, using the R package EpiEstim for the red zone.^5^ Figure 1 shows that incidence in the red zone increased until March 5th, when a steady reduction started.^6^ Coherently, there was a drop in the values of *R*_t_, which decreased from 2 to 0.9 and remained stable afterward. As the incidence rates in the provinces of Milan and Pavia, which we used to approximate the rates in the municipalities of the Lodi province out of the red zone, are among the lowest in the Lombardy Region, our results should be regarded as conservative. Had we applied the rates of other provinces, the estimated reduction in *R*_t_ would have been even larger.

**FIGURE 1:**
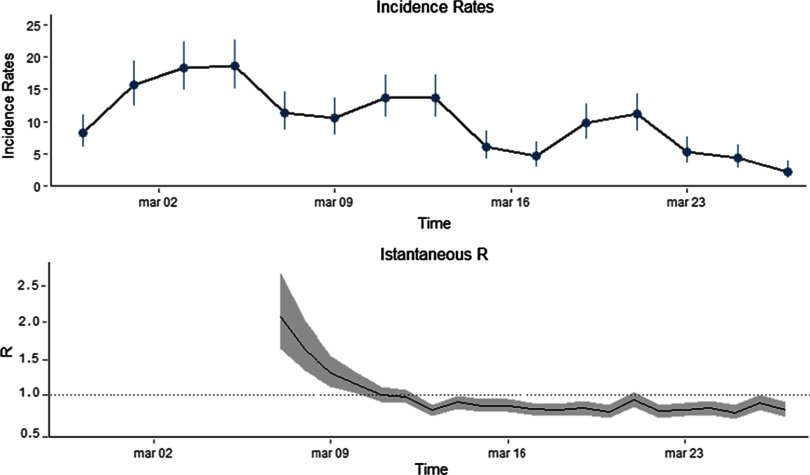
(Upper Panel) Incidence rates (per 10,000 inhabitants) of COVID-19 cases in the red zone from February 28th to March 27th. (Lower Panel) Time-varying reproduction number *R_t_* in the red zone. *R_t_* estimates are based on a 7-day sliding window and assuming a serial interval with a mean of 4.7 days and a standard deviation of 2.9 days.^6^

These results are consistent with what observed in Wuhan Province, China,^5^ and suggest that the effects of a lockdown can be appreciated in approximately 2 wk, a time range that is consistent with the COVID-19 incubation period combined with the delay of the diagnosis after symptoms onset.^1^ Should this be the case, it is plausible to expect a similar reduction in the countries that recently introduced similar measures, if population compliance to the enforced containment measures is similar to that seen in the red zone. These results offer the hope that mitigation measures similar to those issued by the Italian Government can influence the progression of local transmission of COVID-19. They also provide support to the implementation of analogous policies in other countries.

We evaluated the short-term effects of mitigation measures imposed by the Italian government on the first 10 municipalities affected by severe acute respiratory syndrome coronavirus-2 (SARS-CoV-2) spread. Our results suggest that the effects of containment measures can be appreciated after approximately 2 wk.
